# Methicillin Resistance Transfer from *Staphylocccus epidermidis* to Methicillin-Susceptible *Staphylococcus aureus* in a Patient during Antibiotic Therapy

**DOI:** 10.1371/journal.pone.0011841

**Published:** 2010-07-29

**Authors:** Alexander L. A. Bloemendaal, Ellen C. Brouwer, Ad C. Fluit

**Affiliations:** 1 Department of Medical Microbiology, University Medical Center Utrecht, Utrecht, The Netherlands; 2 Department of Surgery, University Medical Center Utrecht, Utrecht, The Netherlands; National Institute of Allergy and Infectious Diseases, National Institutes of Health, United States of America

## Abstract

**Background:**

The *mecA* gene, encoding methicillin resistance in staphylococci, is located on a mobile genetic element called Staphylococcal Cassette Chromosome *mec* (SCC*mec*). Horizontal, interspecies transfer of this element could be an important factor in the dissemination of methicillin-resistant *S. aureus* (MRSA). Previously, we reported the isolation of a closely related methicillin-susceptible *Staphylococcus aureus* (MSSA), MRSA and potential SCC*mec* donor *Staphylococcus epidermidis* isolate from the same patient. Based on fingerprint techniques we hypothesized that the *S. epidermidis* had transferred SCC*mec* to the MSSA to become MRSA. The aim of this study was to show that these isolates form an isogenic pair and that interspecies horizontal SCC*mec* transfer occurred.

**Methodology/Results:**

Whole genome sequencing of both isolates was performed and for the MSSA gaps were closed by conventional sequencing. The SCC*mec* of the *S. epidermidis* was also sequenced by conventional methods. The results show no difference in nucleotide sequence between the two isolates except for the presence of SCC*mec* in the MRSA. The SCC*mec* of the *S. epidermidis* and the MRSA are identical except for a single nucleotide in the *ccrB* gene, which results in a valine to alanine substitution. The main difference with the closely related EMRSA-16 is the presence of SaPI2 encoding toxic shock syndrome toxin and exfoliative toxin A in the MSSA-MRSA pair. No transfer of SCC*mec* from the *S. epidermidis* to the MSSA could be demonstrated *in vitro*.

**Conclusion:**

The MSSA and MRSA form an isogenic pair except for SCC*mec*. This strongly supports our hypothesis that the MRSA was derived from the MSSA by interspecies horizontal transfer of SCC*mec* from *S. epidermidis* O7.1.

## Introduction

Methicillin-resistant *Staphylococcus aureus* (MRSA) is a major cause of nosocomial and community-acquired infections. Resistance to methicillin is a result of the expression of penicillin-binding protein 2a (PBP2a), which causes a low affinity for β-lactam antibiotics. PBP2a is encoded by the *mecA* gene, which is located on a mobile genetic element called Staphylococcal Cassette Chromosome *mec* (SCC*mec*) [1]. To date, several types of SCC*mec* have been described which were recently reclassified [2].

Horizontal transfer of mobile genetic elements contributes to the worldwide dissemination of (multi)-resistant pathogens. Leverstein-van Hall *et al.* described a high rate of interspecies transfer of resistance genes among multidrug resistant Enterobacteriaceae in patients [3]. Berglund *et al.* described the likely transfer of a type V SCC*mec* from methicillin resistant *Staphylococcus haemolyticus* to *S. aureus* in a neonatal intensive care unit [4]. Although this interspecies transfer of SCC*mec* could contribute profoundly to the dissemination of MRSA, evidence is still inconclusive.

Previously, we described the isolation of a successive pair of *mecA* ^−^ and *mecA* ^+^ *S. aureus* and a methicillin resistant *Staphylococcus epidermidis* isolate, which was likely the donor of the SCC*mec*, from a male infant suffering Pierre Robin syndrome [5]. The patient was intubated and mechanically ventilated 4 days after birth because of respiratory insufficiency. A suspected respiratory tract infection was treated with amoxicillin/clavulanic acid. As he became bacteraemic after 3 days the antibiotic regimen was changed to amoxicillin and cefotaxime. When his blood cultures grew *mecA* ^−^ methicillin-susceptible *S. aureus* (MSSA) treatment was changed to flucloxacillin. He seemed to recover, but amoxicillin/clavulanic acid treatment was reinstated for 10 more days on day 32 after the respiratory tract infection had recurred. On day 56, routine cultures from nasal swabs unexpectedly showed a *mecA* ^+^ MRSA. Several strains of *mecA* ^+^ coagulase-negative staphylococci (CNS) were also identified. Pulsed-Field Gel Electrophoresis (PFGE) of the isolates using *Sma*I showed a 40 kb shift of a single DNA fragment between the MSSA and MRSA. Southern hybridization showed that the larger DNA fragment contained the *mecA* gene. The MRSA and MSSA had identical ribotypes and a rare phage type not recorded previously by the Dutch reference center. The PFGE pattern was also not encountered among the profiles of 312 European MRSA isolates. The WKZ-1 and 2 were susceptible for all antibiotics tested except that the MRSA was resistant to β-lactam antibiotics. Southern blotting of MRSA and CNS DNA cut with *Cla*I, *Eco*R1, and *Hin*dIII using a SCC*mec*-specific probe obtained from the MRSA showed identical fingerprints for the MRSA and one of the CNS isolates. From these data we concluded that likely transfer of SCC*mec* from a *S. epidermidis* to a MSSA had occurred [5].

To obtain more definite proof that the *S. epidermidis* was the donor of the SCC*mec*, the whole genome sequences of the MSSA and MRSA were determined as well as the sequence of the SCC*mec* sequence of the *S. epidermidis* isolate.

## Results

For MSSA WKZ-1 a total of 45 contigs larger than 1000 nucleotides was obtained with an average read size of 244 nucleotides and a 23.5-fold coverage, whereas for WKZ-2 a total of 136 contigs larger than 1000 nucleotides were obtained with an average read size of 187 nucleotides and a 9.9-fold coverage. The isolates belong to Sequence Type (ST) 30. MRSA252, which is an EMRSA-16 and has ST36 and belongs to the same Clonal Complex (CC30) was the most closely related strain from which the whole genome was sequenced. Therefore, the sequence of this strain was used as a scaffold to determine the order and orientation of the contigs for further DNA sequencing of the gaps between the contigs. Except for SCC*mec* no difference in the mobile genetic elements of WKZ-1 and WKZ-2 was found.

**Figure 1:**
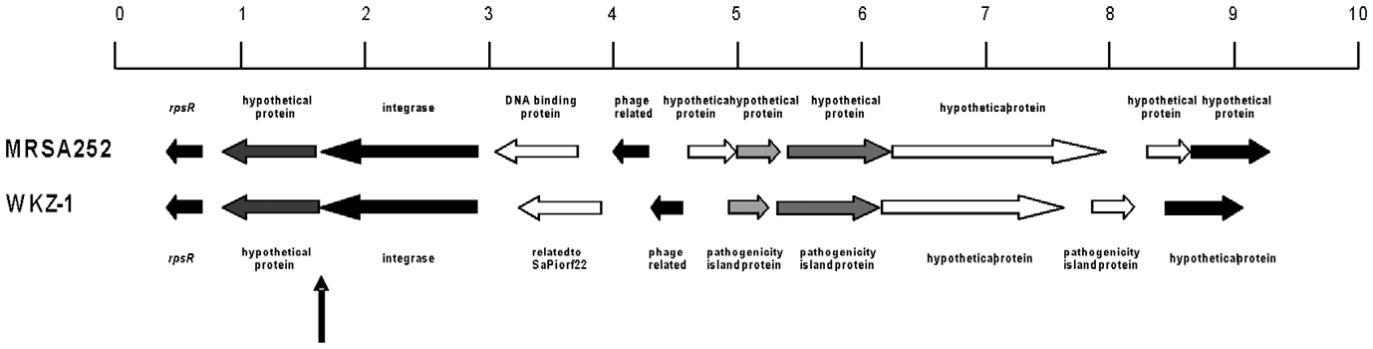
Alignment of the first part of SaPI2 from MRSA252 (top) with WKZ-1. Horizontal arrows indicate open reading frame possibly encoding proteins. White indicates no similarity between the proteins. The increasing greys scale indicates increasing similarity. Black arrows indicate identical sequences. Vertical arrow indicates the boundary between the *S. aureus* chromosome and SaPi2.

The WKZ isolates differ from MRSA252 in a number of mobile genetic elements. The WKZ-2 MRSA contains a SCC*mec* type IV, whereas MRSA252 carries a SCC*mec* type II of 58.8 kb which includes pUB110 encoding bleomycin and kanamycin resistance and Tn*554* encoding resistance to erythromycin and streptomycin. At the position of the *Staphylococcus aureus* Pathogenicity Island 4 (SaPI4) in MRSA252 the WKZ isolates carries a slightly different SaPI (bp 356,242-372,489). It shows differences the presence or absence of open reading frames (orfs) and similarity between homologous genes (Fig. 1). The function of these proteins is unknown, but a BLAST against the GenBank database shows homology with pathogenicity island proteins. In addition, SaPI2 is present in the WKZ isolates (bp 2,116,959-2,131,693) which encodes toxic shock syndrome toxin and exfoliative toxin A. The genomic island νSaα is identical for the WKZ isolates and MRSA252 with two exceptions. One exotoxin gene shows 3 nucleotide deletions leading to a slightly different C-terminal amino acid sequence for this protein. The second dissimilarity, which is in a protein part of a restriction modification system, contains 20 point-mutations which lead to 5 amino acid changes. In addition, the restriction modification system present in genomic island νSaβ of MRSA252 is not functional in the WKZ isolates as the gene encoding the methylase is truncated. Finally, an additional gene for a SD-repeat protein (*sdrD*) is present in the WKZ isolates but not in MRSA252. Both MRSA252 and the WKZ isolates contain the *sdrC* and *bbp* gene, which are also SD-repeat proteins. The latter gene encodes bone sialoprotein binding protein.

**Figure 2:**
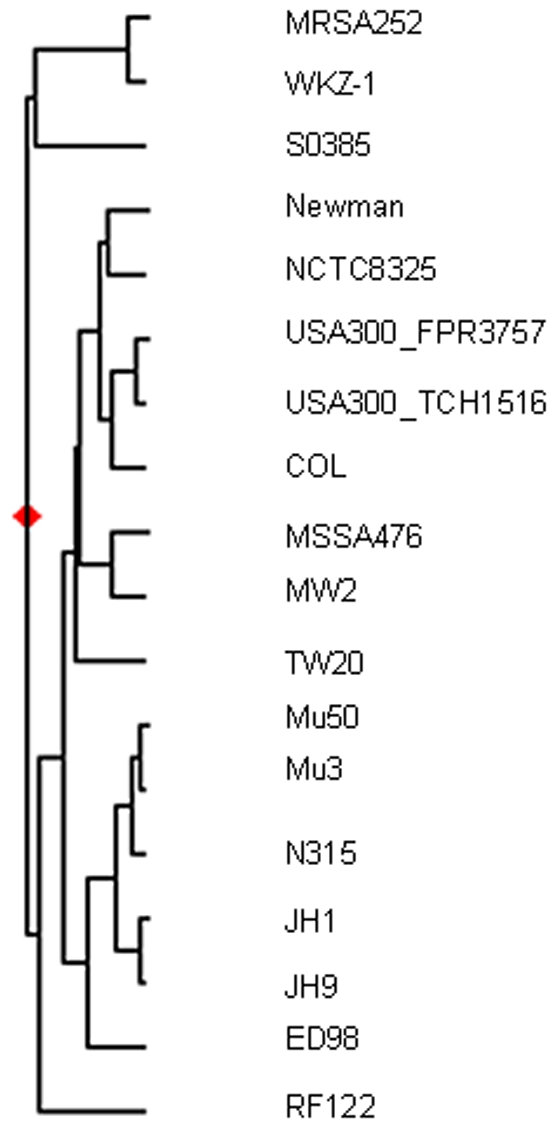
Phylogenetic analysis of WKZ-1 and 17 other *S. aureus* strains for which complete genomes are publicly available.

The WKZ isolates differs in 423 single nucleotide polymorphisms or SNPs from MRSA252 (181 missense mutations, 97 silent mutations 85 mutations at intergenic regions, and 60 indels) in the core genome of *S. aureus*. The number of SNPs in the variable part of the *S. aureus* shared by the 2 isolates was much higher with 1001 SNPs (558 missense mutations, 344 silent mutations 22 mutations at intergenic regions, and 77 indels). Based on these data the dN/dS ratio for the core is approximately 0.6. The close relationship between the WKZ and MRSA252 isolates is further confirmed by a phylogenic analysis based on the sequence of WKZ-1 and 17 other published complete genomes. In this analysis WKZ-1 and MRSA252 cluster together (Fig. 2)

No differences were observed in the sequences of WKZ-1 and WKZ-2, except for the presence of SCC*mec* in the latter isolate. The isolates can therefore be considered isogenic. The SCC*mec* of the proposed donor *S. epidermidis* isolate O7.1 belonged to type IVa. However, the SCC*mec* of WKZ-2 contains an additional sequence that is homologous to the 6987 bp cassette chromosome(SCC)-like element of strain ATCC25923 [6]. This element was already present in WKZ-1. The SCC*mec* IVa sequence of *S. epidermidis* O7.1 is identical to that of the WKZ-2 isolate except for a single point-mutation that resulted in a valine to alanine amino acid substitution in *ccrB*, which encodes a recombinase involved in the excision and integration of SCC*mec*. The lack of differences between WKZ-1 and WKZ-2 and the presence of only one point-mutation between the SCC*mec* elements of the proposed donor *S. epidermidis* and WKZ-2 strongly support our hypothesis that WKZ-2 was derived from WKZ-1 by transfer of SCC*mec* from *S. epidermidis* O7.1.

Resistance to antibiotics was studied. *S. epidermidis* 07.1 was resistant to cefoxitin, ampicillin, penicillin, amoxicillin/clavulanate, and trimethroprim/sulfametoxazol. MSSA WKZ-1 was not resistant to any of the tested antibiotics. WKZ-2 MRSA was resistant only to oxacillin.

Replication of the transfer from *S. epidermidis* to WKZ-1 using *in vitro* filter matings in the presence or absence of β-lactam antibiotics was not successful.

## Discussion

We previously reported the isolation of a closely related MSSA and MRSA and potential SCC*mec* donor *S. epidermidis* isolate from the same patient. Based on fingerprint techniques we hypothesized that the *S. epidermidis* had transferred SCC*mec* to the WKZ-1 MSSA to become the WKZ-2 MRSA [5]. In the present study, we performed whole genome sequencing on both isolates. The results show no difference in nucleotide sequence between the two WKZ isolates except for the presence of SCC*mec* in WKZ-2. A previous study had shown that SCC*mec* of WKZ-2 was bordered by the SCC-like element of *S. aureus* strain ATCC25923, whereas this element was lacking in the *S. epidermidis* strain. The SCC-like element may encode 2 transposases and 3 hypothetical proteins. Sequencing of the SCC*mec* of the *S. epidermidis* showed that it was identical to the SCC*mec* of WKZ-2 except for a single point-mutation. The SCC-like element that is also present in WKZ-2 was also previously shown to be present in WKZ-1 [7]. In the current study we show that the sequences of the SCC-like element are identical in both isolates, except for a single point-mutation in the *ccrB* gene which leads to a valine to alanine substitution in the encoded recombinase.

Apparently, the WKZ strain was able to accept SCC elements on 2 occasions. This strain has the ability to excise these SCC elements either independently or together, but excision at an alternative site was also noted previously [7]. The element could also be excised from the supposed *S. epiderdmidis* donor [7]. A possible cause for the ease of the acceptance of SCC elements is the inactivation of a restriction modification enzyme in νSaβ by the introduction of a frame-shift. In addition, the additional mutations present in the restriction modification system of νSaα may also influence the functionality of this system. Restriction modification systems are a way for bacteria to control the uptake of foreign DNA. The hypothesis that restriction modification systems influence the ability of different *S. aureus* strains to accept SCC element is also supported by data from Waldron and Lindsay [8], who showed that variation in the restriction modification system corresponded with the 10 major *S. aureus* lineages.

Based on the data of this study and data of Nübel *et al*., which indicate that transfer of SCC*mec* into *S. aureus* is not as rare as previously believed [9], [10], we tried to replicate the transfer of SCC*mec* from the *S. epidermidis* O7.1 to WKZ-1 in filter matings. However, these experiments were unsuccessful. This was also the case when the antibiotics used to treat the child were used to induce transfer. This suggests that transfer of SCC*mec* to *S. aureus* requires special circumstances that we still do not understand in contrast to the situation in Enterobacteriaceae where transfer of resistance elements can be easily achieved both *in vitro* and in patients [3].

The WKZ-2 is likely to be more pathogenic than MRSA252, which is a representative of EMRSA-16, as WKZ-2 contains a number of additional virulence factors. Most notably, the WKZ-2 strain contains SaPI2, which encodes both toxic shock syndrome toxin and exfoliative toxin. Especially toxic shock syndrome toxin has been associated with severe disease [11], whereas exfoliative toxin A, which acts as a specific protease has been associated with skin damage [12]. The acquisition of SaPI may also be the result of the impaired restriction modifications systems. So, impaired restriction modification may lead to the emergence of novel antibiotic-resistant strains, but also more pathogenic strains. In addition, the *sdrD* gene is present in WKZ-2 which encodes a putative adhesion protein. This may play a role in tissue specificity of the strain. A difference in a νSaα encoded exotoxin was also observed.

The relatedness of WKZ-1 (ST30) and MRSA252 (ST36) is demonstrated by the relatively low number of SNPs (423 in the *S. aureus* core genome and 1001 in the shared variable genome). In addition, the phylogenetic tree based on the complete genomes of 18 *S. aureus* strains, shows the close relatedness of MRSA252 and the WKZ isolates. The interpretation of the dN/dS ratio of 0.6 is not possible, because it may indicate negative or positive selection or no selection at all in intraspecies analysis of selective pressure [13]. To establish whether particular genes or operons were under selective pressure the presence of multiple mutations in single genes or operons were analyzed. Eight genes and one operon [14] (Table 1) were identified as having more than mutation had multiple mutations. Only of one the genes had only silent mutations. The mutations in DNA topoisomerase I can be explained by differences in fluoroquinolone susceptibility. The very large surface anchored protein is most likely exposed on the outside of the bacterium and might be sensitive to immune pressure. The only operon is involved in the synthesis of the *S. aureus* pigment, a known virulence factor [15], [16], how mutations affect the synthesis in different hosts in unknown. The mutations in the genes for DNA polymerase I, a possible formate dehydrogenase, and a natural resistance associated protein family member are not readily explained, but may represent chance mutations that have not been purified from the strain through selection [13].

**Table 1:** Genes and operons with multiple SNPs between WKZ-1 and MRSA252.

potential function	type of mutation	bp postion in MRSA252
natural resistance associated macrophage protein family protein	N	1,125,728
	N	1,125,731
	N	1,125,792
	N	1,125,806
hypothetical protein	S	1,126,585
	N	1,126,662
DNA topoisomerase I	S	1,279,226
	N	1,280,755
very large surface anchored protein	N	1,503,515
	N	1,505,090
	S	1,508,470
	S	1,514,431
	N	1,521,422
	S	1,529,950
DNA polymerase I	S	1,832,748
	N	1,834,661
putative formate dehydrogenase	S	2,462,696
	N	2,463,997
squalene synthetase	N	2,731,700
squelene desaturase	S	2,732,542
putative glycosyl transeferase	N	2,733,561

N is non-synomous, S is synomous.

In conclusion, the data presented strongly support our hypothesis that interspecies transfer of a SCC*mec* took place, in a patient undergoing antibiotic therapy, from *S. epidermidis* O7.1 to MSSA WKZ-1, to become MRSA WKZ-2. Our hypothesis is that MSSA WKZ-1 is receptive for mobile genetic elements, due to an alteration or impairment of two restriction modification systems. Unfortunately, we were unable to replicate SCC*mec* transfer *in vitro*. This may indicate that the interspecies transfer of SCC*mec* is either relatively rare, or that the *in vitro* circumstances were unfavorable. Alternatively, virulence is the driving force for more frequent recombination. In this hypothesis low frequencies of homologous recombination are maintained and occasionally acquire novel genes that result in virulence by horizontal genetic transfer. The resulting pathogenic lifestyle results in greater exposure to host immune defenses [17]. Selection for such variants results in higher mutation and recombination rates. Nevertheless, the presence of *S. aureus* strains with impaired restriction modification systems may lead to the emergence of novel and highly pathogenic strains.

## Materials and Methods

### Ethics statement

An ethics statement is not required for this work.

### DNA sequencing

The MSSA and MRSA isolates WKZ-1 and WKZ-2 were sequenced by KeyGene (Wageningen, The Netherlands) using 454 sequencing technology. For WKZ-1 the gaps between the contigs with the exception of the rRNA operons were closed by PCR amplification of the missing fragments followed by conventional DNA sequencing. MRSA252 was used as a scaffold. Annotation was performed by JCVI. The sequencing of WKZ-1 has been reported in GenBank under project ID 40253. The SCC*mec* sequence is accessible under no. GQ918137.

Single-nucleotide polymorphism (SNP) analysis was performed using NUCMER [http://mummer.sourceforge.net/] followed by confirmation using PCR amplification and conventional DNA sequencing.

The SCC*mec* of the *S. epidermidis* was sequenced by conventional sequencing of PCR products. PCR amplifications were based on the sequence of the SCC*mec* of WKZ-2. Conventional sequencing was performed by BaseClear (Leiden, The Netherlands).

### *In vitro* SCC*mec* transfer

To select *S. aureus* that may have obtained SCC*mec* from the *S. epidermidis* donor strains during *in vitro* mating experiments the MSSA was made resistant to linezolid by serial transfer, because the *S. epidermidis* isolate was resistant to commonly used antibiotics. Bacteria were mated on nitrocellulose filters. Mating experiments without antibiotics were performed using TSA 5% sheep blood plates (BD Diagnostic Systems, USA). Mating experiments under antibiotic pressure were performed using cloxacillin at concentrations of 0.5, 0.25 and 0.125 µg/mL and cefotaxime at concentrations of 1.0, 0.5, 0.25 µg/mL in LB agar. Conjugants were selected using both linezolid and cloxacillin at 4.0 µg/mL each.

### Antibiograms

To assess antimicrobial resistance of the WKZ-1 MSSA and the *S. epidermidis* 07.1 isolates, the minimal inhibitory concentration (MIC) were determined for a number of clinically relevant antibiotics.
